# Pre-pregnancy maternal exposure to polybrominated and polychlorinated biphenyls and gestational diabetes: a prospective cohort study

**DOI:** 10.1186/s12940-016-0092-5

**Published:** 2016-01-20

**Authors:** Lindsay M. Jaacks, Dana Boyd Barr, Rajeshwari Sundaram, José M. Maisog, Cuilin Zhang, Germaine M. Buck Louis

**Affiliations:** Hubert Department of Global Health, Rollins School of Public Health, Emory University, Claudia Nance Rollins Building 7040-I, 1518 Clifton Rd NE, Atlanta, GA 30322 USA; Department of Environmental Health, Rollins School of Public Health, Emory University, Atlanta, GA USA; Biostatistics and Bioinformatics Branch, Division of Intramural Population Health Research, Eunice Kennedy Shriver National Institute of Child Health and Human Development, Rockville, MD USA; Glotech, Inc, Rockville, MD USA; Division of Intramural Population Health Research, Eunice Kennedy Shriver National Institute of Child Health and Human Development, Rockville, MD USA

**Keywords:** Persistent organic pollutants, Lipids, Diabetes, Pregnancy

## Abstract

**Background:**

While several studies have shown an association between environmental pollutants and diabetes among non-pregnant adults, few studies have prospectively assessed the association among pregnant women. We estimated the association between maternal pre-pregnancy levels of a polybrominated biphenyl (PBB 153) and 36 polychlorinated biphenyls (PCBs) with gestational diabetes (GDM).

**Methods:**

Data are from women (18–40 years) participating in a prospective cohort who achieved pregnancy lasting ≥24 weeks gestation and completed monthly pregnancy journals (*n* = 258). Women were recruited between 2005 and 2007 from 16 counties in Michigan and Texas. Women who ever reported a physician diagnosis of high blood glucose in monthly pregnancy journals were categorized as having gestational diabetes (*n* = 28; 10.9 %). Multivariable binary logistic regression was used to estimate odds ratios (OR) and 95 % confidence intervals (CIs).

**Results:**

There was no association between PBB 153 and GDM or any of the PCB congeners and GDM in unadjusted models. All associations remained non-significant with stepwise adjustment for age and waist-to-height ratio. Only with further adjustment for total serum lipids did the associations become significant, with lower levels of nine PCB congeners associated with GDM: 138, 153, 156, 167, 170, 172, 178, 180, and 194. The adjusted ORs for PCBs 170 and 180 were the strongest: 0.40 (0.18, 0.88) and 0.41 (0.19, 0.87), respectively.

**Conclusions:**

Pre-pregnancy levels of PCBs were not consistently associated with development of GDM in this prospective cohort of U.S. women. Interestingly, we found that although women with GDM had higher mean pre-pregnancy circulating lipid levels compared to women without GDM, they had lower wet weight circulating levels of many PCBs. More research is needed to understand the dynamic fluctuations of PCBs and other persistent organic pollutants between lipid compartments in women preparing to conceive and throughout pregnancy.

**Electronic supplementary material:**

The online version of this article (doi:10.1186/s12940-016-0092-5) contains supplementary material, which is available to authorized users.

## Background

Gestational diabetes (GDM) is common in the United States, affecting as many as 9.2 % of live births [1]. GDM is associated with increased risk of type 2 diabetes in the mother [2] and unfavorable metabolic phenotypes in the offspring [3–8]; thus, GDM prevention is an important goal of larger efforts to address the type 2 diabetes epidemic. Increased maternal age and pre-pregnancy obesity are established risk factors for GDM [9–11]. Only a few studies have explored the role of environmental chemicals in the etiology of GDM. The U.S. Agricultural Health Study found a significant association between self-reported use of agricultural pesticides (but not residential pesticides) during the first trimester of pregnancy and GDM: odds ratio (OR) and 95 % confidence interval (CI), 2.2 (1.5, 3.3) [12]. A study of women in the French West Indies found no significant association between maternal serum chlordecone levels at delivery and GDM recorded in medical records: OR (95 % CI), 0.7 (0.5, 1.1) [13]. A study of pre-pregnancy serum perfluorooctanoic acid in the U.S. general population found a significant positive association with GDM risk: OR (95 % CI), 1.86 (1.14, 3.02) [14].

To our knowledge, no study has evaluated the association between polybrominated biphenyls (PBBs) and GDM, and only a few studies have evaluated the association between PBBs and diabetes among non-pregnant adults. For example, a 25-year follow-up of the Michigan PBB Cohort did not find a significant association between serum PBB levels and incident type 2 diabetes [15]. Similarly, low dose exposure to PBB among individuals without diabetes was not significantly associated with insulin resistance measured 20 years later [16]. In contrast, low dose exposure to PBB in NHANES 2003–2004 (a cross-sectional sample) was significantly associated with type 2 diabetes [17].

While several studies have evaluated the association between polychlorinated biphenyls (PCBs) and type 2 diabetes, to our knowledge, none have evaluated the association with GDM. A recent meta-analysis of six prospective studies (four in the United States, one in Sweden, and one in Taiwan) found that higher levels of total serum PCBs were significantly associated with an increased risk of type 2 diabetes in non-pregnant men and women [18].

Clearly, a significant gap remains in the scientific literature relating environmental chemical exposures and GDM. The objective of this analysis was to estimate the association of pre-pregnancy PBB 153 and 36 PCB congeners with GDM.

## Methods

### Study sample

Data are from a prospective cohort, the Longitudinal Investigation of Fertility and the Environment (LIFE) Study [19]. The sample was recruited between 2005 and 2007 from 16 counties in Michigan and Texas. The Texan Parks and Wildlife Department’s angler database and, in Michigan, a commercially available marketing database, were used to identify individuals to whom recruitment materials should be mailed. Individuals were screened for eligibility via telephone interview within 2 weeks of this mailing. Eligibility criteria included: (1) married or in a committed relationship, (2) aged 18–40 years for women and ≥18 years for men, (3) self-reported menstrual cycles within the range of 21–42 days, (4) no hormonal birth control injections in the past 12 months, and (5) English or Spanish-speaking. Of 51,715 individuals screened via this process, only 1188 (2.3 %) were eligible. Among eligible participants, 501 enrolled in the study and 347 achieved pregnancy of which 258 (74 %) women completed monthly pregnancy journals for their pregnancies lasting ≥24 weeks gestation. This gestational age cut-point is consistent with the recommended time to start universal screening for GDM [20].

Following a baseline study visit, which was conducted by a nurse and assistant at the participants’ home, women were followed daily until a positive pregnancy test and through the first seven post-conception weeks of pregnancy, and then monthly until delivery. Approval for use of human subjects was obtained from all collaborating institutions and all participants provided informed consent.

### Exposure assessment

Laboratory assessment was conducted by the Division of Laboratory Sciences in the National Center for Environmental Health at the Centers for Disease Control and Prevention. Pre-pregnancy, non-fasting blood samples were collected during the baseline study visit into ethylenediaminetetraacetic acid (EDTA) tubes, which were spun down and aliquoted immediately, and the plasma stored at ≤70 °C. The laboratory tested and selected all venipuncture and collection equipment, assuring that they were free of persistent organic pollutants (POPs).

High performance gas chromatography-high resolution mass spectrometry at 10,000 resolution was used to quantify pre-pregnancy serum concentrations of one PBB congener and 36 PCB congeners [21]. An enzymatic summation method was used to quantify serum concentrations of total cholesterol, nonesterified cholesterol, triglycerides, and phospholipids [22]. Total lipid was calculated using the Phillips formula [23].

PBB, PCB, and total lipid values were natural log-transformed (x + 1) and rescaled by their standard deviation to aid interpretation of results. The mean (SD) limit of detection (LOD) across samples was 0.0025 (0.0002) ng/g serum for all PCB congeners except PCB 28 (LOD mean [SD] of 0.0082 [0.0006] ng/g serum) and PCB 52 (LOD mean [SD] of 0.0040 [0.0003] ng/g serum). The LOD mean (SD) for PBB 153 was 0.0026 (0.0005) ng/g serum. We did not substitute concentrations below the limit of detection, as this practice can introduce bias in estimation of human health outcomes [24, 25]. We also made the a priori decision not to sum all 36 PCB congeners given that this data reduction technique assumes that summed components act via the same mechanism and elicit the same effects [26]. We did, however, conduct a sensitivity analysis to evaluate the effects of the sum of dioxin-like PCB congeners (congeners 105, 118, 156, 157, 167, and 189), and the sum of non-dioxin-like PCB congeners (remaining 30 congeners) [27]. For seven PCB congeners (49, 52, 87, 128, 149, 151, and 189), >90 % of samples had levels below the LOD. When these PCB congeners were dichotomized as above versus below the LOD, no significant associations with GDM were observed in either unadjusted or adjusted analyses, thus they were excluded from further analysis.

### Outcome assessment

In monthly pregnancy journals, which were designed to be consistent with recommendations of the American Congress for Obstetricians and Gynecologists for antenatal care including the time of GDM, women were instructed to record whether their obstetrical health care provider told them they had high blood sugar associated with pregnancy. Women were also encouraged to take their pregnancy journal with them to doctor’s appointments. Women who ever reported a physician diagnosis of high blood glucose during pregnancy that was not pre-existing were categorized as having gestational diabetes (*n* = 28; 10.9 %).

### Covariate assessment

Women were also interviewed about their lifestyle and medical/reproductive histories followed by anthropometric assessment (height, weight, and waist and hip circumferences) using an established protocol [28] during the baseline study visit. Pre-pregnancy body mass index (BMI) was calculated as measured weight in kilograms divided by height in meters-squared [29] and categorized as BMI <25.0 kg/m^2^ versus BMI ≥25.0 kg/m^2^ (overweight or obese) [30]. Two markers of visceral adiposity were used: (1) high waist circumference, defined as a waist circumference >88 cm [31, 32] and (2) high waist-to-height ratio, defined as a waist-to-height ratio >0.5 [33].

Gestational weight gain was calculated as the difference between measured pre-pregnancy weight and the last self-reported pregnancy weight from monthly pregnancy journals. Women were classified into three categories based on pre-pregnancy BMI-specific U.S. Institute of Medicine Guidelines [34]: (1) gained less than ideal weight, (2) gained ideal weight, and (3) gained more than ideal weight.

### Statistical analysis

All analyses were conducted using SAS software version 9.4 (SAS Institute, Cary, North Carolina). Markov Chain Monte Carlo methods were implemented to impute missing chemical and lipid data (≤4 % missing, see Additional file 1) using other chemical exposures [35, 36]. A total of 10 multiple imputations were computed. Descriptive statistics were used to explore the distributions of exposures and covariates. Bivariate associations between covariates and GDM were evaluated using chi-square tests and analysis of variance (ANOVA). Bivariate associations between covariates and chemicals were evaluated using ANOVA and correlation coefficients. SAS PROC MIANALYZE was used to combine means and standard deviations from the multiple imputations. Correlation coefficients from the multiple imputations were combined using Fisher’s z transformation [37, 38]. Scatterplots were used to visualize the association between chemicals and lipids stratified by GDM status.

**Fig. 1:**
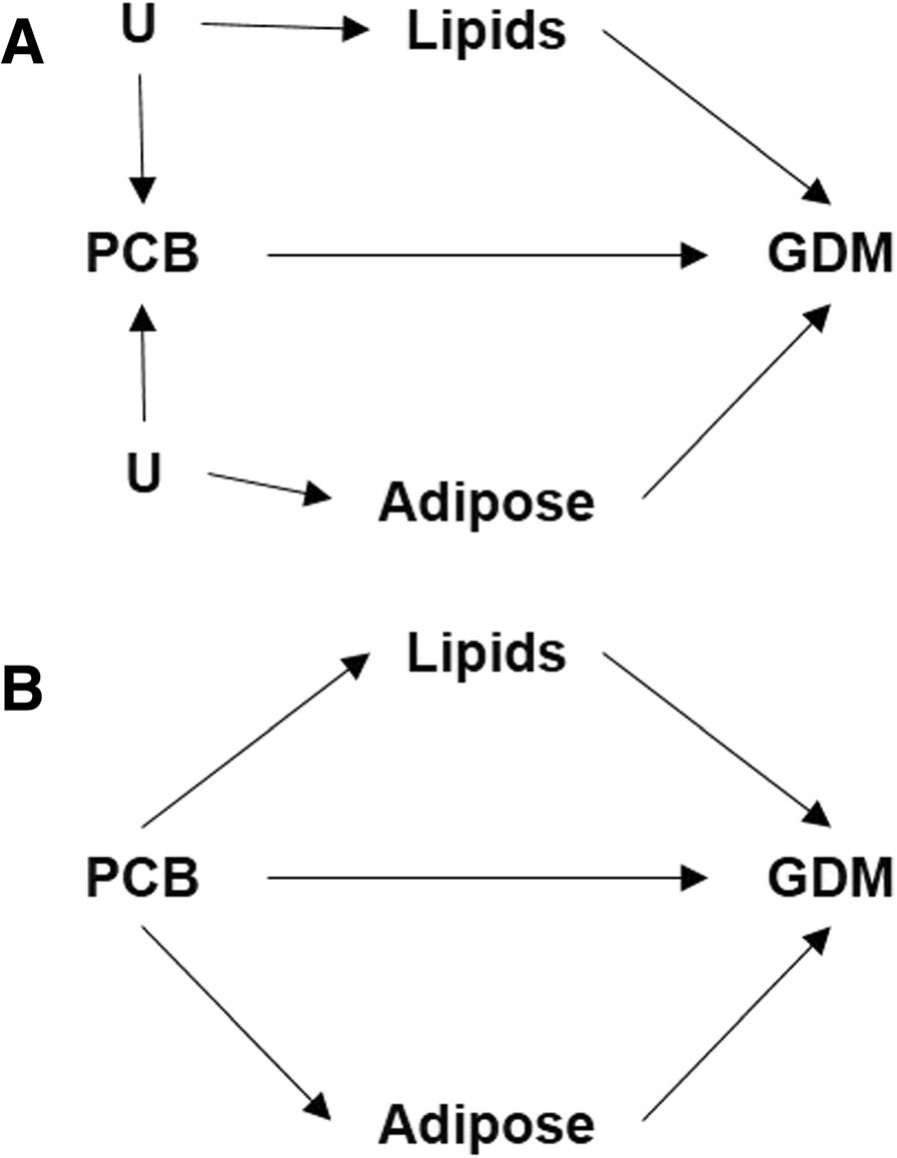
Two potential directed acyclic graphs of the association between pre-pregnancy serum concentrations of polychlorinated biphenyls (PCBs) and gestational diabetes (GDM). Panel a: Assumes that an unknown or unmeasured variable (“U”) causes both circulating lipid levels and circulating PCB levels, and that both circulating lipid levels and circulating PCB levels independently cause the outcome, GDM. Panel b: Assumes that circulating PCB levels cause circulating lipid levels, which in turn cause the outcome, GDM

Multivariable binary logistic regression was used to estimate ORs and 95 % CIs for the association between PBB 153 and each of the 36 PCB congeners with GDM. Separate models were run for each chemical or congener. SAS PROC MIANALYZE was used to calculate the average of the 10 complete-data estimates from the multiple imputations. The variance of this estimate was the sum of the between- and within-imputation variances. Two models were reported for PBB 153 and each of the PCB congeners: unadjusted and adjusted for maternal pre-pregnancy age, waist-to-height ratio, and total serum lipids, all specified continuously. These two models reflect two hypothesized directed acyclic graphs (Fig. 1). The first directed acyclic graph (Fig. 1a) assumes that an unmeasured variable (“U”) causes both circulating lipid levels and circulating PCB levels, and that both circulating lipid levels and circulating PCB levels independently cause the outcome, GDM. This is the traditional confounding structure and the effect estimate is least biased when adjusted for total serum lipids [39]. The second directed acyclic graph (Fig. 1b) assumes that circulating PCB levels cause circulating lipid levels, which in turn cause the outcome, GDM. In this case, the effect estimate is least biased when unadjusted for total serum lipids [39]. Other confounders were selected according to the following three properties: associated with the exposure (i.e. chemicals), associated with the disease (i.e. GDM), and not on the causal pathway between exposure and disease [40]. As this was an exploratory analysis, *p*-values were not adjusted for multiple testing.

## Results

Compared to women who did not develop GDM, women who developed GDM were more likely to report a weight gain of ≥20 kg since adolescence; having themselves been a low-birth-weight baby; a history of GDM during past pregnancies; and gaining less than the recommended amount of weight during the current pregnancy (Table 1). There were no significant differences in age, parity/gravidity, or smoking/tobacco use, though women who developed GDM tended to be >25 years old and a race/ethnicity other than non-Hispanic white. With respect to pre-pregnancy anthropometrics, women who developed GDM tended to have greater visceral adiposity, estimated using waist-to-height ratio: 78.6 % of GDM women were classified as having high waist-to-height ratios compared to only 60.0 % of non-GDM women. However, there was no statistically significant difference in BMI status: 53.6 % of GDM women were classified as overweight or obese compared to 45.2 % of non-GDM women.

**Table 1:** Characteristics of participants according to gestational diabetes (GDM) status

	Non-GDM	GDM	*P*-value^b^
	(*n* = 230)	(*n* = 28)	
Pre-pregnancy age (years)	29.6 (3.8)	30.2 (2.9)	0.42
Pre-pregnancy age category (%)			
Age ≤25 years	14.4 (33)	3.6 (1)	0.11
Age >25 years	85.7 (197)	96.4 (27)	
Non-fasting serum lipids (mg/dl)^a^	607.1 (113.6)	678.7 (122.7)	0.0009
Pre-pregnancy BMI (kg/m^2^)	26.1 (6.4)	27.0 (4.6)	0.47
Pre-pregnancy BMI status (%)			
<25 kg/m^2^	54.8 (126)	46.4 (13)	0.40
≥25 kg/m^2^	45.2 (104)	53.6 (15)	
Pre-pregnancy waist circumference (cm)	87.8 (14.8)	90.2 (13.2)	0.41
Pre-pregnancy waist circumference status (%)			
≤88 cm	60.9 (140)	57.1 (16)	0.70
>88 cm	39.1 (90)	42.9 (12)	
Pre-pregnancy waist-to-height ratio status (%)			
≤0.5	40.0 (92)	21.4 (6)	0.06
>0.5	60.0 (138)	78.6 (22)	
Parity and gravidity (%)			
Never pregnant	41.3 (95)	35.7 (10)	0.85
Pregnant without live birth	7.0 (16)	7.1 (2)	
Pregnant with previous birth	51.7 (119)	57.1 (16)	
Race/ethnicity (%)			
Non-Hispanic white	85.7 (197)	75.0 (21)	0.14
Other	14.4 (33)	25.0 (7)	
Pre-pregnancy smoking status (%)			
Yes	6.5 (15)	7.1 (2)	0.90
No	93.5 (215)	92.9 (26)	
Weight gain from 15–19 years old to pre-pregnancy (%)			
<10 kg	46.1 (106)	32.1 (9)	0.05
10–19.9 kg	31.7 (73)	25.0 (7)	
≥20 kg	22.2 (51)	42.9 (12)	
Pregnancy weight gain (%)^c^			
Gained recommended weight	28.2 (59)	35.7 (10)	0.05
Gained < recommended weight	30.6 (64)	46.4 (13)	
Gained > recommended weight	41.2 (86)	17.9 (5)	
Maternal birth weight (%)			
Low (<2000 g)	1.7 (4)	14.3 (4)	0.001
Normal (2000–4000 g)	89.6 (206)	82.1 (23)	
High (>4000 g)	8.7 (20)	3.6 (1)	
Maternal history of GDM (%)			
Yes	0.9 (2)	14.3 (4)	<0.0001
No	99.1 (228)	85.7 (24)	

Values are mean (SD) or % (*n*)

^a^Estimated from the Phillips 1989 equation

^b^ *P*-value from chi-square test for categorical variables and analysis of variance for continuous variables

^c^Gestational weight gain status defined according to the U.S. Institute of Medicine Guidelines as: pre-pregnancy BMI <18.5 kg/m^2^, 11.3–15.9 kg; pre-pregnancy BMI ≥18.5 and <25 kg/m^2^, 12.7–18.1 kg; pre-pregnancy BMI ≥25 and <30 kg/m^2^, 6.8–11.3 kg; pre-pregnancy BMI ≥30 kg/m^2^, 5.0–9.1 kg

There was no association between PBB 153 and GDM or any of the PCB congeners and GDM in unadjusted models (Table 2). All associations remained non-significant with stepwise adjustment for age and waist-to-height ratio (data not shown). Only with further adjustment for total serum lipids did the associations become significant, with lower levels of nine PCB congeners associated with GDM: 138, 153, 156, 167, 170, 172, 178, 180, and 194. The adjusted ORs (95 % CI) for PCBs 170 and 180 were the strongest: 0.40 (0.18, 0.88) and 0.41 (0.19, 0.87), respectively. The adjusted OR (95 % CI) for the sum of dioxin-like PCB congeners was 0.65 (0.37, 1.15) and for the sum of non-dioxin-like PCB congeners it was 0.37 (0.13, 1.04). When models were adjusted for individual lipid components rather than total lipids, results were attenuated towards the null but remained significant for PCBs 156, 167, 170, 172, and 180 (data not shown). Results were also consistent in a complete-case analysis and with additional adjustment for weight gain during current pregnancy, history of gestational diabetes, weight gain since age 15–19 years, or study site (data not shown).

**Table 2:** Association of pre-pregnancy serum polybrominated and polychlorinated biphenyl levels with gestational diabetes (*n* = 258)

	Unadjusted^a^	Adjusted^b^
Polybrominated biphenyl (rescaled, natural-log transformed)		
153	0.73 (0.37, 1.43)	0.68 (0.31, 1.49)
Polychlorinated biphenyls (rescaled, natural-log transformed)		
28	0.91 (0.42, 1.98)	0.90 (0.24, 3.31)
44	0.83 (0.20, 3.43)	0.88 (0.24, 3.23)
66	0.96 (0.58, 1.60)	0.96 (0.49, 1.87)
74	0.89 (0.44, 1.81)	0.52 (0.13, 2.06)
99	1.00 (0.67, 1.48)	0.78 (0.48, 1.29)
101	1.05 (0.75, 1.48)	1.00 (0.69, 1.47)
105	1.09 (0.76, 1.57)	0.88 (0.57, 1.36)
110	0.88 (0.54, 1.44)	0.82 (0.46, 1.46)
114	0.69 (0.40, 1.21)	0.53 (0.28, 1.00)
118	1.02 (0.69, 1.50)	0.81 (0.51, 1.29)
138	0.75 (0.46, 1.24)	**0.53 (0.29, 0.99)**
146	0.73 (0.43, 1.22)	0.53 (0.28, 1.02)
153	0.70 (0.41, 1.21)	**0.48 (0.24, 0.98)**
156	0.57 (0.32, 1.02)	**0.42 (0.21, 0.87)**
157	0.70 (0.43, 1.13)	0.57 (0.32, 1.03)
167	0.57 (0.31, 1.06)	**0.42 (0.21, 0.84)**
170	0.61 (0.33, 1.12)	**0.40 (0.18, 0.88)**
172	0.60 (0.34, 1.09)	**0.44 (0.23, 0.86)**
177	0.73 (0.31, 1.69)	0.46 (0.17, 1.25)
178	0.66 (0.36, 1.18)	**0.50 (0.25, 0.98)**
180	0.59 (0.32, 1.08)	**0.41 (0.19, 0.87)**
183	0.91 (0.56, 1.50)	0.65 (0.31, 1.35)
187	0.68 (0.34, 1.33)	0.48 (0.21, 1.09)
194	0.68 (0.42, 1.12)	**0.50 (0.27, 0.95)**
195	0.76 (0.47, 1.23)	0.63 (0.37, 1.07)
196	0.79 (0.48, 1.30)	0.60 (0.33, 1.11)
201	0.75 (0.44, 1.27)	0.62 (0.33, 1.17)
206	0.91 (0.58, 1.41)	0.72 (0.41, 1.27)
209	1.09 (0.75, 1.59)	0.92 (0.61, 1.41)

Values are odds ratios (95 % confidence intervals) from multivariable binary logistic regression with no gestational diabetes as the referent category. *P*-values <0.05 are bolded

^a^Corresponds to directed acyclic graph from Fig. 1, Panel a

^b^Adjusted for total serum lipids estimated from the Phillips 1989 equation, age, and waist-to-height ratio, all specified continuously. Corresponds to directed acyclic graph from Fig. 1 , Panel b

**Fig. 2:**
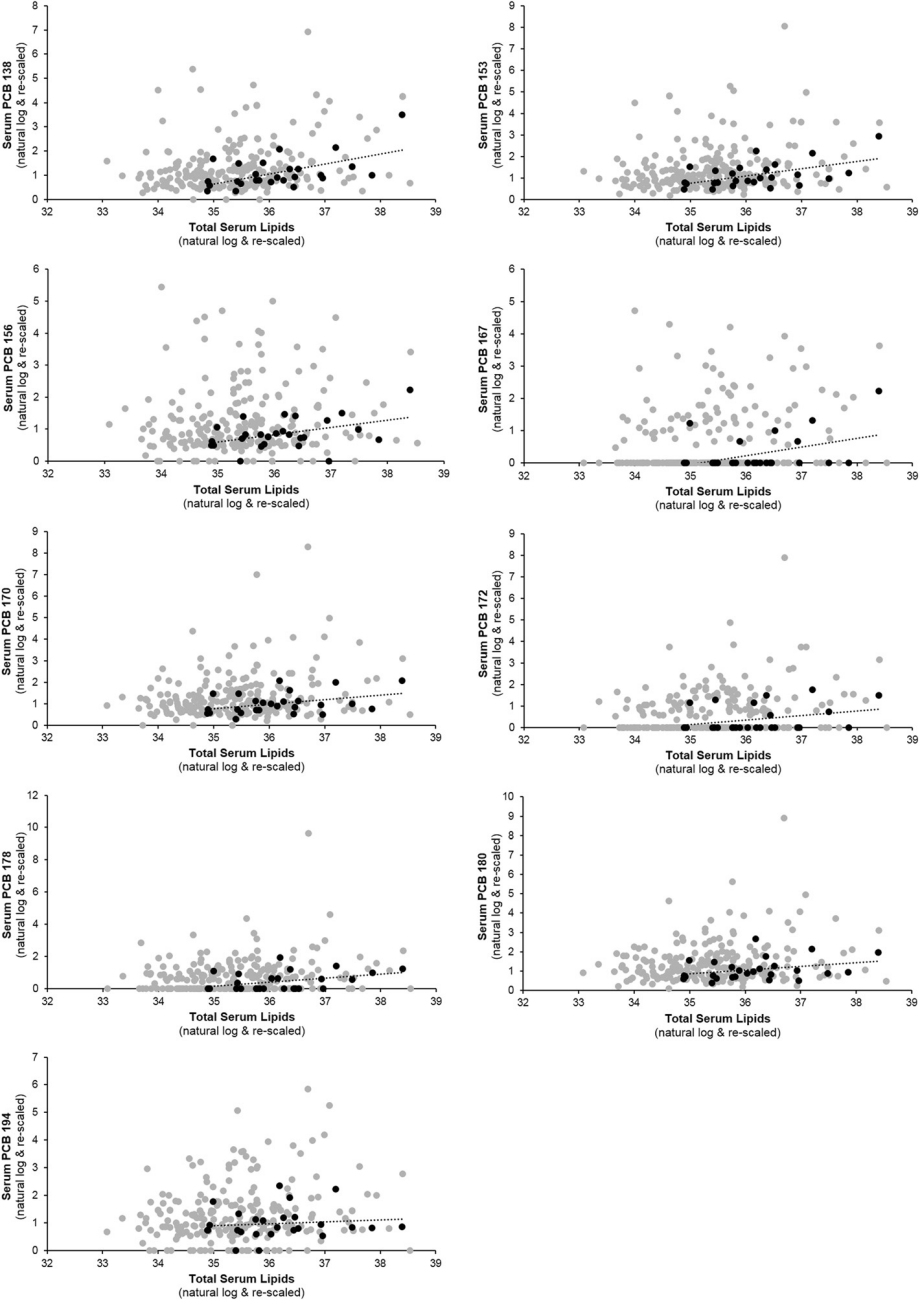
Scatterplot of select pre-pregnancy serum polychlorinated biphenyl (PCB) congeners and total serum lipids for women with (black circles) and without (gray circles) gestational diabetes

Women who went on to develop GDM had higher pre-pregnancy levels of serum lipids: mean (SD) of 678.7 (122.7) mg/dl compared to 607.1 (113.6) mg/dl among women who did not develop GDM. When the association between lipids and select PCBs was plotted according to GDM status, while a correlation between variables was clearly evident among women with GDM, it was shifted relative to women without GDM such that lipids were higher and PCBs lower (Fig. 2).

Several PCB congeners were positively associated with both age and serum lipids including: 99, 105, 118, 138, 146, 153, and all PCBs >167 except PCB 201 (Table 3). Several PCBs also tended to be negatively associated with BMI and waist-to-height ratio, including congeners 156, 157, 180, and 209.

**Table 3:** Correlation between pre-pregnancy serum polybrominated and polychlorinated biphenyl levels and age, body mass index (BMI), waist-to-height ratio (WHR), and total serum lipids (*n* = 258)

	Age (years)	BMI (kg/m^2^)	WHR	Lipids^a^
Polybrominated biphenyl (rescaled, natural-log transformed)				
153	**0.13 (0.03)**	0.01 (0.89)	−0.04 (0.50)	−0.03 (0.59)
Polychlorinated biphenyls (rescaled, natural-log transformed)				
28	−0.03 (0.69)	0.07 (0.26)	0.07 (0.28)	−0.04 (0.55)
44	−0.02 (0.73)	0.07 (0.28)	0.06 (0.33)	−0.05 (0.41)
66	−0.02 (0.78)	0.07 (0.26)	0.07 (0.27)	−0.02 (0.71)
74	0.07 (0.25)	0.06 (0.31)	0.05 (0.46)	0.04 (0.58)
99	**0.23 (0.0003)**	0.02 (0.73)	0.00 (0.99)	**0.19 (0.004)**
101	0.04 (0.55)	0.08 (0.19)	0.10 (0.10)	0.06 (0.31)
105	**0.20 (0.001)**	0.06 (0.32)	0.08 (0.22)	**0.23 (0.0004)**
110	0.06 (0.36)	0.07 (0.26)	0.09 (0.17)	0.01 (0.83)
114	**0.18 (0.003)**	−0.02 (0.75)	0.00 (0.94)	0.09 (0.18)
118	**0.25 (0.0001)**	0.03 (0.59)	0.04 (0.57)	**0.21 (0.001)**
138	**0.27 (<.0001)**	−0.03 (0.66)	−0.03 (0.64)	**0.20 (0.002)**
146	**0.29 (<.0001)**	−0.05 (0.38)	−0.05 (0.47)	**0.15 (0.02)**
153	**0.28 (<.0001)**	−0.08 (0.18)	−0.07 (0.30)	**0.18 (0.005)**
156	**0.19 (0.002)**	**−0.17 (0.007)**	**−0.14 (0.03)**	0.07 (0.25)
157	**0.21 (0.0006)**	**−0.15 (0.01)**	−0.13 (0.05)	0.03 (0.68)
167	**0.26 (<.0001)**	−0.01 (0.88)	−0.02 (0.77)	**0.13 (0.04)**
170	**0.27 (<.0001)**	−0.11 (0.07)	**−**0.10 (0.12)	**0.17 (0.007)**
172	**0.27 (<.0001)**	0.00 (0.99)	0.00 (0.99)	**0.19 (0.004)**
177	**0.17 (0.007)**	0.02 (0.69)	0.03 (0.60)	**0.15 (0.02)**
178	**0.22 (0.0003)**	−0.06 (0.34)	−0.04 (0.51)	**0.15 (0.02)**
180	**0.30 (<.0001)**	−0.12 (0.05)	−0.12 (0.07)	**0.15 (0.02)**
183	**0.22 (0.0004)**	0.04 (0.49)	0.04 (0.49)	**0.20 (0.002)**
187	**0.24 (0.0001)**	0.00 (0.97)	−0.01 (0.92)	**0.15 (0.02)**
194	**0.38 (<.0001)**	−0.10 (0.10)	**−0.13 (0.03)**	**0.13 (0.04)**
195	**0.27 (<.0001)**	−0.04 (0.53)	−0.06 (0.34)	**0.16 (0.01)**
196	**0.36 (<.0001)**	0.00 (0.98)	−0.04 (0.48)	**0.15 (0.02)**
201	**0.34 (<.0001)**	−0.05 (0.42)	−0.09 (0.18)	0.08 (0.21)
206	**0.39 (<.0001)**	−0.02 (0.76)	−0.08 (0.22)	0.13 (0.05)
209	**0.45 (<.0001)**	−0.11 (0.08)	**−0.15 (0.02)**	**0.19 (0.003)**

Values are correlation coefficient (*p*-value). *P*-values <0.05 are bolded

^a^Estimated from the Phillips 1989 equation. Re-scaled and natural-log transformed

**Fig. 3:**
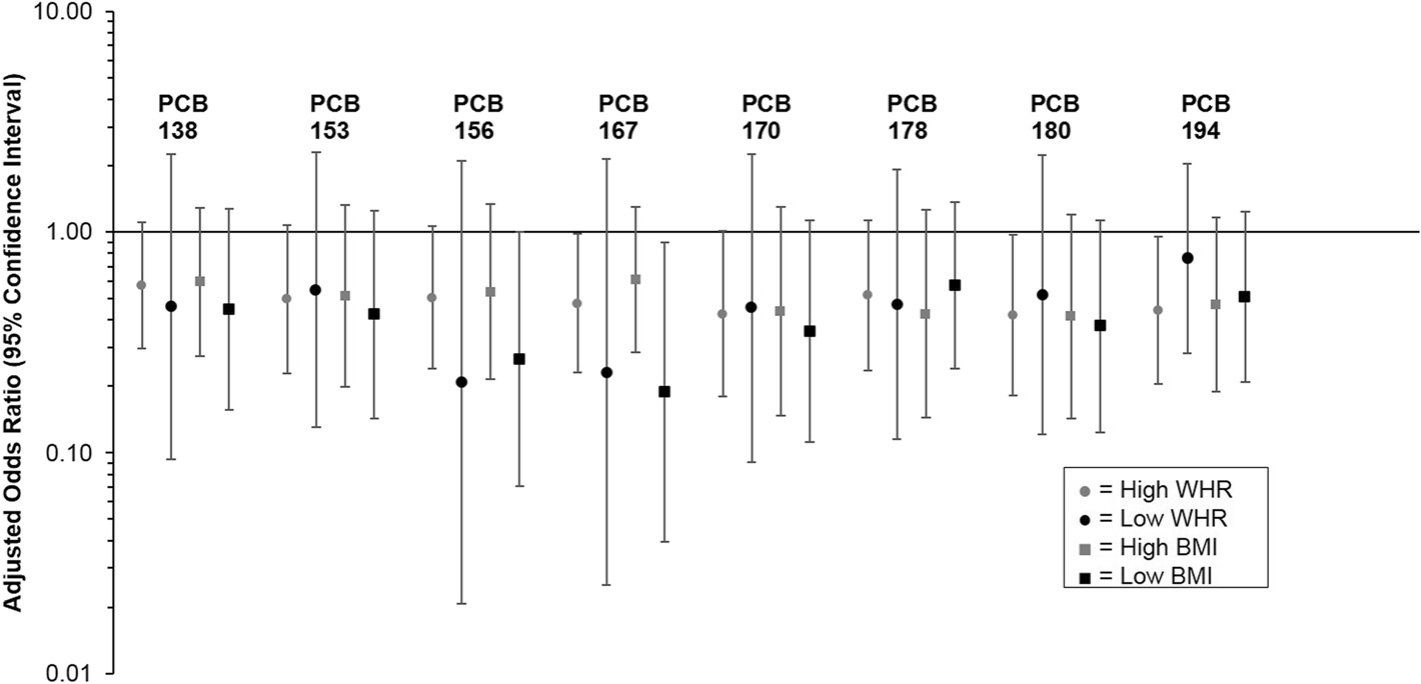
Association between select pre-pregnancy serum polychlorinated biphenyl (PCB) congeners and gestational diabetes stratified by waist-to-height ratio (WHR ≤0.5 versus WHR >0.5) and by body mass index (BMI <25.0 kg/m^2^ versus BMI ≥25.0 kg/m^2^). Values are odds ratios and 95 % confidence intervals from multivariable binary logistic regression, adjusted for age and total serum lipids estimated from the Phillips 1989 equation

**Fig. 4:**
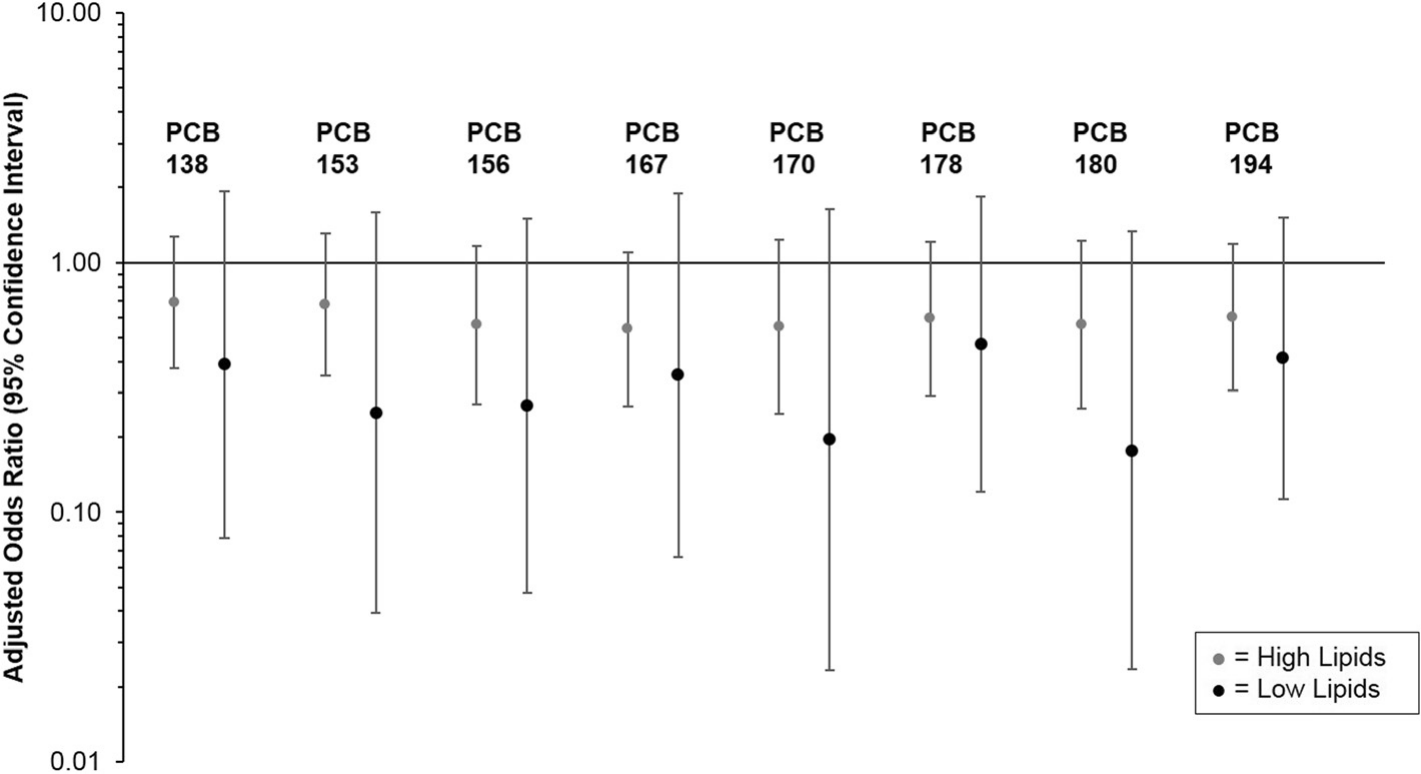
Association between select pre-pregnancy serum polychlorinated biphenyl (PCB) congeners and gestational diabetes stratified by total serum lipids estimated from the Phillips 1989 equation (total serum lipids < median [599.6 mg/dl] versus total serum lipids ≥ median [599.6 mg/dl]). Values are odds ratios and 95 % confidence intervals from multivariable binary logistic regression, adjusted for age and waist-to-height ratio

When models were stratified by visceral adiposity status (high versus normal waist-to-height ratio) and BMI status, differences in associations between groups were only slight and all confidence intervals were overlapping making it difficult to conclude anything about effect modification by body fat (Fig. 3). When models were stratified by lipid status (above versus below the median for the entire sample population), and then adjusted for age and waist-to-height ratio, the association was stronger among those with lower lipids consistently across PCB congeners, but again, all confidence intervals were overlapping (Fig. 4).

## Discussion

Pre-pregnancy levels of PCBs were not consistently associated with GDM in this prospective cohort of U.S. women. Interestingly, we found that although women who developed GDM had higher mean pre-pregnancy circulating lipid levels compared to women who did not develop GDM, they had lower circulating levels of PCBs. Thus, when multivariable models were adjusted for lipids, women who developed GDM had significantly lower pre-pregnancy levels of circulating PCBs (congeners 138, 153, 156, 167, 170, 172, 178, 180, and 194). Because lipids are an important risk factor for the outcome, GDM, careful consideration of the method of lipid adjustment is warranted. If some unmeasured factor affects both circulating PCB and lipid levels, then the least biased models are those adjusting for total serum lipids [39]. However, if the opposite is true and circulating PCBs affect circulating lipids, as causal pathway analysis suggests [41], then the most unbiased models are those that do not adjust for circulating lipids [39]-all of these associations were null in our study.

Previous studies have also reported that serum lipid levels in pregnancy were significantly associated with GDM risk, for instance, women who develop GDM have higher serum triglyceride levels early on in pregnancy than women who do not develop GDM [42–44]. Given the higher level of circulating total lipids among women with GDM in our sample, we expected that levels of the POPs, PBB 153 and PCBs, would be higher because in this sample and in previous studies of non-pregnant adults [45], circulating lipid levels are positively correlated with circulating POPs. However, the levels of PCBs were lower among women with GDM compared to those without GDM. Thus, it may be the case that women who developed GDM sequestered PCBs to a greater extent in other lipid compartments such as adipose tissue, and thus have lower levels of circulating PCBs.

Studies generally assume that in fasting individuals, serum samples can be used to monitor POPs because POPs are equally distributed in all lipid compartments [46]. It is also usually assumed that adjustment for serum lipids in non-fasting individuals can mirror these fasting equilibrium conditions [47]. However, recent studies have cast doubt on the robustness of these assumptions [39, 48]. For example, a small (*n* = 7) study comparing PCB concentrations in serum lipids, visceral fat, and subcutaneous fat, found a “reasonably good correlation” between serum PCBs and subcutaneous fat PCBs for half of participants, but only a weak correlation for the other half [48]. They also reported a tendency toward higher levels of highly chlorinated PCBs in visceral fat compared to subcutaneous fat in half of participants, but in the other participants they found similar levels in these compartments or higher levels in subcutaneous compared to visceral fat [48]. A more recent, larger study (*n* = 189) also reported higher levels of POPs in visceral compared to subcutaneous adipose tissue, but did not assess circulating levels and did not assess PCBs [49]. An operative cohort study of women aged 18–44 years found biological-medium-specific effects of POPs on endometriosis: for example, PCB 151 in omentum fat was significantly associated with increased risk of endometriosis, but not PCB 151 in serum [50]. Clearly more research is needed to test the robustness of common assumptions that serum levels of PCBs reflect levels found in other lipid compartments.

Related to this, we found that women who developed GDM had higher levels of visceral adiposity, as measured using waist-to-height ratio, relative to women who did not develop GDM. This may partially explain the lower circulating levels of PCBs, particularly the more highly chlorinated PCBs. Studies among non-pregnant adults have previously reported an inverse association between circulating levels of highly chlorinated PCBs and visceral and subcutaneous adipose tissue [51–53].

We did not find an association between PBB 153 and GDM, which is consistent with two other prospective analyses that did not find an association between PBBs and type 2 diabetes or insulin resistance [15, 16]. One previous study in obese Japanese adults reported significantly lower levels of PCB 163/164 among individuals with type 2 diabetes (OR adjusted for sex, age, BMI, and total lipids 0.77 [0.62, 0.95]) [54]. They also found lower levels of PCB 153, though it was not statistically significant (0.95 [0.90, 1.00]) [54]. In contrast, a recent meta-analysis of total PCB levels (sum of PCB congeners) in non-pregnant adults found a significant positive association with risk of type 2 diabetes [18]. Further research is needed to disentangle the effects of specific PCB congeners versus PCB congener mixtures on diabetes risk.

Most research on the biological mechanisms underlying the association between PCBs and diabetes has focused on the dioxin-like PCB congeners. Interestingly, when the sum of dioxin-like PCB congeners and the sum of non-dioxin-like PCB congeners were evaluated, the effect was stronger–though not statistically significant-for the non-dioxin-like PCB congers. This is consistent with our results for individual congeners. Nonetheless, without adjustment for serum lipids, all associations were null. Remillard and Bunce [55] have speculated that null associations between PCBs and glucose disorders may relate to the opposing effects of commonly used herbicides such as 2,4-dichlorophenoxyacetic acid, which cause peroxisome proliferation [56, 57], and the potential antagonistic properties of dioxin-like compounds on peroxisome proliferator-activated receptors via activation of the aryl hydrocarbon receptor [58]. Future research should focus on the etiological mechanisms underlying potential effects of non-dioxin-like PCB congeners on glucose metabolism, and on the biological effects of differential partitioning of PCBs across lipid compartments, including adipose tissue and circulating lipid components.

A limitation of this study was that GDM was self-reported. The prevalence of GDM in this cohort was 10.9 % compared to 8.1 % as reported by women participating in the 2007–2008 Pregnancy Risk Assessment Monitoring System (PRAMS) when asked if they had “high blood sugar (diabetes) that started during this (most recent) pregnancy” [1]. Our participants were slightly older than the PRAMS cohort: 86.8 % versus 67.4 % >25 years old, respectively [1]. There are also significant differences between states in the prevalence of self-reported GDM: in the 2010 PRAMS survey, which included Texas but not Michigan, the prevalence of self-reported GDM was 10.0 % in Texas compared to 8.7 % overall [1]. While we did not validate self-reported GDM against medical records, previous studies have found good agreement between the two methods. For example, a validation study of the PRAMS questionnaire conducted in New York City (NYC) and Vermont in 2009 comparing self-reported GDM to medical record data reported a sensitivity of 88.4 % in NYC and 82.0 % in Vermont, specificity of 95.0 % in NYC and 97.1 % in Vermont, and positive predictive value of 49.5 % in both sites [59]. While we recognize that it is possible that our estimate of GDM is an overestimate, it is unlikely that diagnosis or reporting of GDM are associated with the exposure (chemical levels) and, therefore, this is unlikely a major source of bias.

Other limitations beyond those associated with our observational design and use of self-reported GDM include potential confounding by other POPs such as organochlorine pesticides that may also be correlated with PCBs, and residual confounding. In addition, given that this was an exploratory analysis, we did not adjust our p-values for multiple testing so cautious interpretation of results is needed.

## Conclusions

To our knowledge, no study has evaluated the association between pre-pregnancy PBB or PCB concentrations and GDM. More studies are needed to improve our understanding of the associations of these POPs and this important pregnancy outcome given the strong association between GDM and future metabolic disease risk. These should focus on improving our understanding of the dynamic fluctuations of PCBs between lipid compartments in women preparing to conceive and throughout pregnancy.

## Additional file

Additional file 1: Table S1. Summary of pre-pregnancy serum polybrominated and polychlorinated biphenyl levels. **Table S2.** Correlation between polychlorinated biphenyl (PCB) congeners. (DOCX 45 kb)

## Abbreviations

ANOVA: analysis of variance
BMI: body mass index
CI: confidence interval
EDTA: ethylenediaminetetraacetic acid
GDM: gestational diabetes
LOD: limit of detection
LIFE: longitudinal investigation of fertility and the environment
NYC: New York City
OR: odds ratio
POPs: persistent organic pollutants
PBBs: polybrominated biphenyls
PCBs: polychlorinated biphenyls
PRAMS: pregnancy risk assessment monitoring system
