# Supplementary material for: Pre-pregnancy maternal exposure to polybrominated and polychlorinated biphenyls and gestational diabetes: a prospective cohort study
Source: Environ Health. 2016 Jan 20;15:11. doi: 10.1186/s12940-016-0092-5 (PMC4721055; doi:10.1186/s12940-016-0092-5)
Supplement: Additional file 1: Table S1. — Summary of pre-pregnancy serum polybrominated and polychlorinated biphenyl levels. Table S2. Correlation between polychlorinated biphenyl (PCB) congeners. (DOCX 45 kb) [file 12940_2016_92_MOESM1_ESM.docx]

| **Additional file 1 Table S1**. Summary of pre-pregnancy serum polybrominated and polychlorinated biphenyl levels. | | | | |
| --- | --- | --- | --- | --- |
|  | **Missing**  n | **LOD^1^**  mean (SD)  ng/g serum | **<LOD**  % (n) | **Median (Min, Max)**  ng/g serum |
| *Polybrominated biphenyl*  *(rescaled, natural-log transformed)* | | |  |  |
| 153 | 2 | 0.0026 (0.0005) | 9.38 (24) | 0.0068 (0.0000, 0.2317) |
| *Polychlorinated biphenyls*  *(rescaled, natural-log transformed)* | | |  |  |
| 28 | 2 | 0.0082 (0.0006) | 66.80 (171) | 0.0062 (-0.0033, 5.3363) |
| 44 | 11 | 0.0025 (0.0002) | 85.43 (211) | 0.0013 (0.0000, 0.2630) |
| 49 | 11 | 0.0025 (0.0002) | 97.57 (241) | 0.0003 (-0.0002, 0.0948) |
| 52 | 5 | 0.0040 (0.0003) | 96.44 (244) | 0.0007 (-0.0006, 0.2581) |
| 66 | 2 | 0.0025 (0.0002) | 39.06 (100) | 0.0029 (0.0000, 0.6454) |
| 74 | 2 | 0.0025 (0.0002) | 0 (0) | 0.0138 (0.0029, 0.5226) |
| 87 | 2 | 0.0025 (0.0002) | 92.58 (237) | 0.0000 (0.0000, 0.0139) |
| 99 | 5 | 0.0025 (0.0002) | 1.58 (4) | 0.0098 (0.0000, 0.0625) |
| 101 | 2 | 0.0025 (0.0002) | 73.05 (187) | 0.0016 (0.0000, 0.0451) |
| 105 | 2 | 0.0025 (0.0002) | 25.78 (66) | 0.0033 (0.0000, 0.0238) |
| 110 | 5 | 0.0025 (0.0002) | 88.93 (225) | 0.0009 (0.0000, 0.0194) |
| 114 | 2 | 0.0025 (0.0002) | 89.84 (230) | 0.0000 (0.0000, 0.0126) |
| 118 | 2 | 0.0025 (0.0002) | 0.39 (1) | 0.0159 (0.0000, 0.1013) |
| 128 | 2 | 0.0025 (0.0002) | 97.27 (249) | 0.0000 (0.0000, 0.0097) |
| 138 | 2 | 0.0025 (0.0002) | 1.17 (3) | 0.0300 (0.0000, 0.2233) |
| 146 | 10 | 0.0025 (0.0002) | 18.55 (46) | 0.0050 (0.0000, 0.0434) |
| 149 | 9 | 0.0025 (0.0002) | 96.79 (241) | 0.0000 (0.0000, 0.0706) |
| 151 | 2 | 0.0025 (0.0002) | 98.05 (251) | 0.0000 (0.0000, 0.0899) |
| 153 | 2 | 0.0025 (0.0002) | 0 (0) | 0.0423 (0.0064, 0.3752) |
| 156 | 2 | 0.0025 (0.0002) | 11.33 (29) | 0.0054 (0.0000, 0.0337) |
| 157 | 2 | 0.0025 (0.0002) | 76.95 (197) | 0.0012 (0.0000, 0.0097) |
| 167 | 2 | 0.0025 (0.0002) | 77.34 (198) | 0.0000 (0.0000, 0.0100) |
| 170 | 2 | 0.0025 (0.0002) | 1.56 (4) | 0.0127 (0.0000, 0.1053) |
| 172 | 2 | 0.0025 (0.0002) | 79.30 (203) | 0.0000 (0.0000, 0.0153) |
| 177 | 2 | 0.0025 (0.0002) | 56.64 (145) | 0.0022 (0.0000, 0.0740) |
| 178 | 2 | 0.0025 (0.0002) | 62.50 (160) | 0.0017 (0.0000, 0.0316) |
| 180 | 2 | 0.0025 (0.0002) | 0 (0) | 0.0320 (0.0028, 0.2840) |
| 183 | 2 | 0.0025 (0.0002) | 28.91 (74) | 0.0038 (0.0000, 0.0820) |
| 187 | 6 | 0.0025 (0.0002) | 6.75 (17) | 0.0104 (0.0000, 0.1773) |
| 189 | 2 | 0.0025 (0.0002) | 98.05 (251) | 0.0000 (0.0000, 0.0045) |
| 194 | 2 | 0.0025 (0.0002) | 14.06 (36) | 0.0064 (0.0000, 0.0380) |
| 195 | 2 | 0.0025 (0.0002) | 74.61 (191) | 0.0000 (0.0000, 0.0151) |
| 196 | 2 | 0.0025 (0.0002) | 7.81 (20) | 0.0068 (0.0000, 0.0618) |
| 201 | 2 | 0.0025 (0.0002) | 12.50 (32) | 0.0063 (0.0000, 0.0617) |
| 206 | 2 | 0.0025 (0.0002) | 27.73 (71) | 0.0034 (0.0000, 0.0277) |
| 209 | 2 | 0.0025 (0.0002) | 77.34 (198) | 0.0016 (0.0000, 0.0089) |
| ^1^Average limit of detection (LOD) across all participants (each sample has its own LOD). | | | | |

| **Additional file 1 Table S2**. Correlation between PCB congeners. | | | | | | | |
| --- | --- | --- | --- | --- | --- | --- | --- |
| Pearson correlation coefficient  P-value  N | PCB 28 | PCB 44 | PCB 66 | PCB 74 | PCB 99 | PCB 101 | PCB 105 |
| PCB 28 |  | **0.99702 <.0001 247** | **0.99765 <.0001 256** | **0.94466 <.0001 256** | **0.33208 <.0001 253** | **0.61125 <.0001 256** | **0.19900 0.0014 256** |
| PCB 44 | **0.99702 <.0001 247** |  | **0.99389 <.0001 247** | **0.94005 <.0001 247** | **0.33219 <.0001 247** | **0.62091 <.0001 247** | **0.19063 0.0026 247** |
| PCB 66 | **0.99765 <.0001 256** | **0.99389 <.0001 247** |  | **0.95450 <.0001 256** | **0.36644 <.0001 253** | **0.62636 <.0001 256** | **0.24062 0.0001 256** |
| PCB 74 | **0.94466 <.0001 256** | **0.94005 <.0001 247** | **0.95450 <.0001 256** |  | **0.56819 <.0001 253** | **0.60298 <.0001 256** | **0.43395 <.0001 256** |
| PCB 99 | **0.33208 <.0001 253** | **0.33219 <.0001 247** | **0.36644 <.0001 253** | **0.56819 <.0001 253** |  | **0.36125 <.0001 253** | **0.85163 <.0001 253** |
| PCB 101 | **0.61125 <.0001 256** | **0.62091 <.0001 247** | **0.62636 <.0001 256** | **0.60298 <.0001 256** | **0.36125 <.0001 253** |  | **0.27366 <.0001 256** |
| PCB 105 | **0.19900 0.0014 256** | **0.19063 0.0026 247** | **0.24062 0.0001 256** | **0.43395 <.0001 256** | **0.85163 <.0001 253** | **0.27366 <.0001 256** |  |
| PCB 110 | **0.58343 <.0001 253** | **0.60167 <.0001 247** | **0.59025 <.0001 253** | **0.58665 <.0001 253** | **0.40183 <.0001 253** | **0.76759 <.0001 253** | **0.39293 <.0001 253** |
| PCB 114 | 0.06554 0.2962 256 | 0.07096 0.2666 247 | 0.08713 0.1646 256 | **0.28562 <.0001 256** | **0.68307 <.0001 253** | **0.14208 0.0230 256** | **0.58578 <.0001 256** |
| PCB 118 | **0.16366 0.0087 256** | **0.15666 0.0137 247** | **0.20494 0.0010 256** | **0.42270 <.0001 256** | **0.89217 <.0001 253** | **0.28871 <.0001 256** | **0.95530 <.0001 256** |
| PCB 138 | 0.02725 0.6643 256 | 0.02815 0.6598 247 | 0.05564 0.3753 256 | **0.26208 <.0001 256** | **0.77422 <.0001 253** | **0.35377 <.0001 256** | **0.64451 <.0001 256** |
| PCB 146 | 0.02305 0.7179 248 | 0.02760 0.6712 239 | 0.05063 0.4273 248 | **0.21880 0.0005 248** | **0.68414 <.0001 245** | **0.41562 <.0001 248** | **0.58577 <.0001 248** |
| PCB 153 | 0.00311 0.9606 256 | 0.00362 0.9548 247 | 0.02988 0.6342 256 | **0.22187 0.0003 256** | **0.69082 <.0001 253** | **0.38177 <.0001 256** | **0.56789 <.0001 256** |
| PCB 156 | -0.02981 0.6350 256 | -0.02644 0.6792 247 | -0.01183 0.8506 256 | **0.18708 0.0027 256** | **0.60336 <.0001 253** | **0.14210 0.0230 256** | **0.51435 <.0001 256** |
| PCB 157 | -0.04739 0.4503 256 | -0.03948 0.5369 247 | -0.02912 0.6428 256 | **0.16646 0.0076 256** | **0.56984 <.0001 253** | 0.03135 0.6176 256 | **0.47402 <.0001 256** |
| PCB 167 | -0.02727 0.6641 256 | -0.02537 0.6916 247 | 0.00016 0.9980 256 | **0.19137 0.0021 256** | **0.66367 <.0001 253** | **0.21651 0.0005 256** | **0.61291 <.0001 256** |
| PCB 170 | -0.00830 0.8949 256 | -0.00753 0.9062 247 | 0.01139 0.8561 256 | **0.17502 0.0050 256** | **0.50554 <.0001 253** | **0.33322 <.0001 256** | **0.39772 <.0001 256** |
| PCB 172 | 0.01276 0.8391 256 | 0.00936 0.8837 247 | 0.02979 0.6352 256 | **0.14981 0.0165 256** | **0.34829 <.0001 253** | **0.33624 <.0001 256** | **0.26744 <.0001 256** |
| PCB 177 | 0.00727 0.9079 256 | 0.00816 0.8985 247 | 0.02807 0.6548 256 | 0.11108 0.0761 256 | **0.34107 <.0001 253** | **0.62294 <.0001 256** | **0.28208 <.0001 256** |
| PCB 178 | 0.01245 0.8429 256 | 0.01622 0.7997 247 | 0.02960 0.6374 256 | **0.14693 0.0187 256** | **0.42507 <.0001 253** | **0.46205 <.0001 256** | **0.29647 <.0001 256** |
| PCB 180 | -0.01214 0.8468 256 | -0.01197 0.8515 247 | 0.00692 0.9122 256 | **0.16243 0.0092 256** | **0.48195 <.0001 253** | **0.35375 <.0001 256** | **0.37146 <.0001 256** |
| PCB 183 | 0.00631 0.9200 256 | 0.00846 0.8947 247 | 0.02672 0.6705 256 | **0.12433 0.0469 256** | **0.40506 <.0001 253** | **0.55785 <.0001 256** | **0.30247 <.0001 256** |
| PCB 187 | -0.00193 0.9756 252 | -0.00333 0.9588 243 | 0.01791 0.7773 252 | **0.13069 0.0382 252** | **0.38234 <.0001 249** | **0.51275 <.0001 252** | **0.33106 <.0001 252** |
| PCB 194 | -0.01836 0.7700 256 | -0.01482 0.8167 247 | -0.00352 0.9552 256 | 0.11951 0.0562 256 | **0.37445 <.0001 253** | **0.23331 0.0002 256** | **0.26742 <.0001 256** |
| PCB 195 | 0.02981 0.6350 256 | 0.03527 0.5812 247 | 0.04711 0.4530 256 | **0.17190 0.0058 256** | **0.39275 <.0001 253** | **0.36742 <.0001 256** | **0.30079 <.0001 256** |
| PCB 196 | -0.02015 0.7483 256 | -0.01976 0.7574 247 | -0.00198 0.9748 256 | 0.12251 0.0502 256 | **0.41353 <.0001 253** | **0.36203 <.0001 256** | **0.31065 <.0001 256** |
| PCB 201 | -0.02034 0.7460 256 | -0.01755 0.7837 247 | -0.00581 0.9263 256 | 0.10261 0.1014 256 | **0.34853 <.0001 253** | **0.29803 <.0001 256** | **0.25276 <.0001 256** |
| PCB 206 | -0.02495 0.6911 256 | -0.02248 0.7252 247 | -0.01168 0.8525 256 | 0.11040 0.0779 256 | **0.36137 <.0001 253** | 0.09361 0.1353 256 | **0.31300 <.0001 256** |
| PCB 209 | -0.01316 0.8340 256 | -0.01275 0.8419 247 | 0.00109 0.9862 256 | 0.11900 0.0572 256 | **0.36251 <.0001 253** | 0.01612 0.7975 256 | **0.32349 <.0001 256** |

| **Supplemental Table 2**. Correlation between PCB congeners. | | | | | | | |
| --- | --- | --- | --- | --- | --- | --- | --- |
| Pearson correlation coefficient  P-value  N | PCB 110 | PCB 114 | PCB 118 | PCB 138 | PCB 146 | PCB 153 | PCB 156 |
| PCB 28 | **0.58343 <.0001 253** | 0.06554 0.2962 256 | **0.16366 0.0087 256** | 0.02725 0.6643 256 | 0.02305 0.7179 248 | 0.00311 0.9606 256 | -0.02981 0.6350 256 |
| PCB 44 | **0.60167 <.0001 247** | 0.07096 0.2666 247 | **0.15666 0.0137 247** | 0.02815 0.6598 247 | 0.02760 0.6712 239 | 0.00362 0.9548 247 | -0.02644 0.6792 247 |
| PCB 66 | **0.59025 <.0001 253** | 0.08713 0.1646 256 | **0.20494 0.0010 256** | 0.05564 0.3753 256 | 0.05063 0.4273 248 | 0.02988 0.6342 256 | -0.01183 0.8506 256 |
| PCB 74 | **0.58665 <.0001 253** | **0.28562 <.0001 256** | **0.42270 <.0001 256** | **0.26208 <.0001 256** | **0.21880 0.0005 248** | **0.22187 0.0003 256** | **0.18708 0.0027 256** |
| PCB 99 | **0.40183 <.0001 253** | **0.68307 <.0001 253** | **0.89217 <.0001 253** | **0.77422 <.0001 253** | **0.68414 <.0001 245** | **0.69082 <.0001 253** | **0.60336 <.0001 253** |
| PCB 101 | **0.76759 <.0001 253** | **0.14208 0.0230 256** | **0.28871 <.0001 256** | **0.35377 <.0001 256** | **0.41562 <.0001 248** | **0.38177 <.0001 256** | **0.14210 0.0230 256** |
| PCB 105 | **0.39293 <.0001 253** | **0.58578 <.0001 256** | **0.95530 <.0001 256** | **0.64451 <.0001 256** | **0.58577 <.0001 248** | **0.56789 <.0001 256** | **0.51435 <.0001 256** |
| PCB 110 |  | **0.19184 0.0022 253** | **0.33183 <.0001 253** | **0.28394 <.0001 253** | **0.29007 <.0001 245** | **0.28668 <.0001 253** | **0.18015 0.0040 253** |
| PCB 114 | **0.19184 0.0022 253** |  | **0.66420 <.0001 256** | **0.65349 <.0001 256** | **0.60222 <.0001 248** | **0.60594 <.0001 256** | **0.70691 <.0001 256** |
| PCB 118 | **0.33183 <.0001 253** | **0.66420 <.0001 256** |  | **0.74443 <.0001 256** | **0.68447 <.0001 248** | **0.67265 <.0001 256** | **0.60403 <.0001 256** |
| PCB 138 | **0.28394 <.0001 253** | **0.65349 <.0001 256** | **0.74443 <.0001 256** |  | **0.91031 <.0001 248** | **0.95814 <.0001 256** | **0.79137 <.0001 256** |
| PCB 146 | **0.29007 <.0001 245** | **0.60222 <.0001 248** | **0.68447 <.0001 248** | **0.91031 <.0001 248** |  | **0.92958 <.0001 248** | **0.75262 <.0001 248** |
| PCB 153 | **0.28668 <.0001 253** | **0.60594 <.0001 256** | **0.67265 <.0001 256** | **0.95814 <.0001 256** | **0.92958 <.0001 248** |  | **0.82825 <.0001 256** |
| PCB 156 | **0.18015 0.0040 253** | **0.70691 <.0001 256** | **0.60403 <.0001 256** | **0.79137 <.0001 256** | **0.75262 <.0001 248** | **0.82825 <.0001 256** |  |
| PCB 157 | 0.08313 0.1875 253 | **0.74218 <.0001 256** | **0.56997 <.0001 256** | **0.65763 <.0001 256** | **0.60458 <.0001 248** | **0.65260 <.0001 256** | **0.85554 <.0001 256** |
| PCB 167 | **0.16615 0.0081 253** | **0.67470 <.0001 256** | **0.70467 <.0001 256** | **0.81475 <.0001 256** | **0.78279 <.0001 248** | **0.79025 <.0001 256** | **0.68801 <.0001 256** |
| PCB 170 | **0.20653 0.0010 253** | **0.44983 <.0001 256** | **0.49670 <.0001 256** | **0.85025 <.0001 256** | **0.83797 <.0001 248** | **0.92861 <.0001 256** | **0.80202 <.0001 256** |
| PCB 172 | **0.16055 0.0105 253** | **0.36562 <.0001 256** | **0.35592 <.0001 256** | **0.71121 <.0001 256** | **0.73016 <.0001 248** | **0.75342 <.0001 256** | **0.56603 <.0001 256** |
| PCB 177 | **0.31780 <.0001 253** | **0.25528 <.0001 256** | **0.37666 <.0001 256** | **0.69635 <.0001 256** | **0.74335 <.0001 248** | **0.75369 <.0001 256** | **0.41215 <.0001 256** |
| PCB 178 | **0.28005 <.0001 253** | **0.42487 <.0001 256** | **0.40309 <.0001 256** | **0.76584 <.0001 256** | **0.80270 <.0001 248** | **0.81938 <.0001 256** | **0.59366 <.0001 256** |
| PCB 180 | **0.22519 0.0003 253** | **0.43273 <.0001 256** | **0.47572 <.0001 256** | **0.83860 <.0001 256** | **0.83943 <.0001 248** | **0.92544 <.0001 256** | **0.77360 <.0001 256** |
| PCB 183 | **0.29318 <.0001 253** | **0.29380 <.0001 256** | **0.39742 <.0001 256** | **0.76690 <.0001 256** | **0.76013 <.0001 248** | **0.81400 <.0001 256** | **0.45578 <.0001 256** |
| PCB 187 | **0.30598 <.0001 249** | **0.31415 <.0001 252** | **0.42017 <.0001 252** | **0.74994 <.0001 252** | **0.79361 <.0001 244** | **0.82614 <.0001 252** | **0.52653 <.0001 252** |
| PCB 194 | **0.12403 0.0488 253** | **0.34810 <.0001 256** | **0.35712 <.0001 256** | **0.61951 <.0001 256** | **0.64927 <.0001 248** | **0.70233 <.0001 256** | **0.62922 <.0001 256** |
| PCB 195 | **0.23966 0.0001 253** | **0.38529 <.0001 256** | **0.39241 <.0001 256** | **0.68719 <.0001 256** | **0.67093 <.0001 248** | **0.72964 <.0001 256** | **0.55873 <.0001 256** |
| PCB 196 | **0.20292 0.0012 253** | **0.28534 <.0001 256** | **0.39908 <.0001 256** | **0.66179 <.0001 256** | **0.66722 <.0001 248** | **0.71700 <.0001 256** | **0.51481 <.0001 256** |
| PCB 201 | **0.17066 0.0065 253** | **0.30954 <.0001 256** | **0.33482 <.0001 256** | **0.56698 <.0001 256** | **0.61694 <.0001 248** | **0.63567 <.0001 256** | **0.49905 <.0001 256** |
| PCB 206 | 0.08096 0.1993 253 | **0.28519 <.0001 256** | **0.36511 <.0001 256** | **0.43271 <.0001 256** | **0.41769 <.0001 248** | **0.45919 <.0001 256** | **0.39992 <.0001 256** |
| PCB 209 | 0.00913 0.8851 253 | **0.35314 <.0001 256** | **0.39126 <.0001 256** | **0.47475 <.0001 256** | **0.47606 <.0001 248** | **0.49010 <.0001 256** | **0.44933 <.0001 256** |

| **Supplemental Table 2**. Correlation between PCB congeners. | | | | | | | |
| --- | --- | --- | --- | --- | --- | --- | --- |
| Pearson correlation coefficient  P-value  N | PCB 157 | PCB 167 | PCB 170 | PCB 172 | PCB 177 | PCB 178 | PCB 180 |
| PCB 28 | -0.04739 0.4503 256 | -0.02727 0.6641 256 | -0.00830 0.8949 256 | 0.01276 0.8391 256 | 0.00727 0.9079 256 | 0.01245 0.8429 256 | -0.01214 0.8468 256 |
| PCB 44 | -0.03948 0.5369 247 | -0.02537 0.6916 247 | -0.00753 0.9062 247 | 0.00936 0.8837 247 | 0.00816 0.8985 247 | 0.01622 0.7997 247 | -0.01197 0.8515 247 |
| PCB 66 | -0.02912 0.6428 256 | 0.00016 0.9980 256 | 0.01139 0.8561 256 | 0.02979 0.6352 256 | 0.02807 0.6548 256 | 0.02960 0.6374 256 | 0.00692 0.9122 256 |
| PCB 74 | **0.16646 0.0076 256** | **0.19137 0.0021 256** | **0.17502 0.0050 256** | **0.14981 0.0165 256** | 0.11108 0.0761 256 | **0.14693 0.0187 256** | **0.16243 0.0092 256** |
| PCB 99 | **0.56984 <.0001 253** | **0.66367 <.0001 253** | **0.50554 <.0001 253** | **0.34829 <.0001 253** | **0.34107 <.0001 253** | **0.42507 <.0001 253** | **0.48195 <.0001 253** |
| PCB 101 | 0.03135 0.6176 256 | **0.21651 0.0005 256** | **0.33322 <.0001 256** | **0.33624 <.0001 256** | **0.62294 <.0001 256** | **0.46205 <.0001 256** | **0.35375 <.0001 256** |
| PCB 105 | **0.47402 <.0001 256** | **0.61291 <.0001 256** | **0.39772 <.0001 256** | **0.26744 <.0001 256** | **0.28208 <.0001 256** | **0.29647 <.0001 256** | **0.37146 <.0001 256** |
| PCB 110 | 0.08313 0.1875 253 | **0.16615 0.0081 253** | **0.20653 0.0010 253** | **0.16055 0.0105 253** | **0.31780 <.0001 253** | **0.28005 <.0001 253** | **0.22519 0.0003 253** |
| PCB 114 | **0.74218 <.0001 256** | **0.67470 <.0001 256** | **0.44983 <.0001 256** | **0.36562 <.0001 256** | **0.25528 <.0001 256** | **0.42487 <.0001 256** | **0.43273 <.0001 256** |
| PCB 118 | **0.56997 <.0001 256** | **0.70467 <.0001 256** | **0.49670 <.0001 256** | **0.35592 <.0001 256** | **0.37666 <.0001 256** | **0.40309 <.0001 256** | **0.47572 <.0001 256** |
| PCB 138 | **0.65763 <.0001 256** | **0.81475 <.0001 256** | **0.85025 <.0001 256** | **0.71121 <.0001 256** | **0.69635 <.0001 256** | **0.76584 <.0001 256** | **0.83860 <.0001 256** |
| PCB 146 | **0.60458 <.0001 248** | **0.78279 <.0001 248** | **0.83797 <.0001 248** | **0.73016 <.0001 248** | **0.74335 <.0001 248** | **0.80270 <.0001 248** | **0.83943 <.0001 248** |
| PCB 153 | **0.65260 <.0001 256** | **0.79025 <.0001 256** | **0.92861 <.0001 256** | **0.75342 <.0001 256** | **0.75369 <.0001 256** | **0.81938 <.0001 256** | **0.92544 <.0001 256** |
| PCB 156 | **0.85554 <.0001 256** | **0.68801 <.0001 256** | **0.80202 <.0001 256** | **0.56603 <.0001 256** | **0.41215 <.0001 256** | **0.59366 <.0001 256** | **0.77360 <.0001 256** |
| PCB 157 |  | **0.64405 <.0001 256** | **0.58106 <.0001 256** | **0.42908 <.0001 256** | **0.24055 0.0001 256** | **0.44124 <.0001 256** | **0.57053 <.0001 256** |
| PCB 167 | **0.64405 <.0001 256** |  | **0.67736 <.0001 256** | **0.61008 <.0001 256** | **0.52844 <.0001 256** | **0.60513 <.0001 256** | **0.66483 <.0001 256** |
| PCB 170 | **0.58106 <.0001 256** | **0.67736 <.0001 256** |  | **0.79885 <.0001 256** | **0.71885 <.0001 256** | **0.80377 <.0001 256** | **0.97770 <.0001 256** |
| PCB 172 | **0.42908 <.0001 256** | **0.61008 <.0001 256** | **0.79885 <.0001 256** |  | **0.71143 <.0001 256** | **0.79937 <.0001 256** | **0.82315 <.0001 256** |
| PCB 177 | **0.24055 0.0001 256** | **0.52844 <.0001 256** | **0.71885 <.0001 256** | **0.71143 <.0001 256** |  | **0.79791 <.0001 256** | **0.75596 <.0001 256** |
| PCB 178 | **0.44124 <.0001 256** | **0.60513 <.0001 256** | **0.80377 <.0001 256** | **0.79937 <.0001 256** | **0.79791 <.0001 256** |  | **0.83752 <.0001 256** |
| PCB 180 | **0.57053 <.0001 256** | **0.66483 <.0001 256** | **0.97770 <.0001 256** | **0.82315 <.0001 256** | **0.75596 <.0001 256** | **0.83752 <.0001 256** |  |
| PCB 183 | **0.28879 <.0001 256** | **0.58476 <.0001 256** | **0.77873 <.0001 256** | **0.76039 <.0001 256** | **0.93449 <.0001 256** | **0.82340 <.0001 256** | **0.80948 <.0001 256** |
| PCB 187 | **0.34550 <.0001 252** | **0.57267 <.0001 252** | **0.80718 <.0001 252** | **0.78991 <.0001 252** | **0.91163 <.0001 252** | **0.86416 <.0001 252** | **0.86912 <.0001 252** |
| PCB 194 | **0.50634 <.0001 256** | **0.50016 <.0001 256** | **0.79875 <.0001 256** | **0.69525 <.0001 256** | **0.56865 <.0001 256** | **0.66439 <.0001 256** | **0.85437 <.0001 256** |
| PCB 195 | **0.43339 <.0001 256** | **0.57171 <.0001 256** | **0.76890 <.0001 256** | **0.68386 <.0001 256** | **0.67168 <.0001 256** | **0.71499 <.0001 256** | **0.80513 <.0001 256** |
| PCB 196 | **0.37424 <.0001 256** | **0.50116 <.0001 256** | **0.75578 <.0001 256** | **0.71270 <.0001 256** | **0.71211 <.0001 256** | **0.72223 <.0001 256** | **0.83310 <.0001 256** |
| PCB 201 | **0.38480 <.0001 256** | **0.44905 <.0001 256** | **0.67254 <.0001 256** | **0.65277 <.0001 256** | **0.61866 <.0001 256** | **0.66995 <.0001 256** | **0.76831 <.0001 256** |
| PCB 206 | **0.35283 <.0001 256** | **0.34739 <.0001 256** | **0.48761 <.0001 256** | **0.43292 <.0001 256** | **0.34311 <.0001 256** | **0.41657 <.0001 256** | **0.56730 <.0001 256** |
| PCB 209 | **0.42005 <.0001 256** | **0.46141 <.0001 256** | **0.53190 <.0001 256** | **0.48239 <.0001 256** | **0.26077 <.0001 256** | **0.41654 <.0001 256** | **0.55623 <.0001 256** |

| **Supplemental Table 2**. Correlation between PCB congeners. | | | | | | | | |
| --- | --- | --- | --- | --- | --- | --- | --- | --- |
| Pearson correlation coefficient  P-value  N | PCB 183 | PCB 187 | PCB 194 | PCB 195 | PCB 196 | PCB 201 | PCB 206 | PCB 209 |
| PCB 28 | 0.00631 0.9200 256 | -0.00193 0.9756 252 | -0.01836 0.7700 256 | 0.02981 0.6350 256 | -0.02015 0.7483 256 | -0.02034 0.7460 256 | -0.02495 0.6911 256 | -0.01316 0.8340 256 |
| PCB 44 | 0.00846 0.8947 247 | -0.00333 0.9588 243 | -0.01482 0.8167 247 | 0.03527 0.5812 247 | -0.01976 0.7574 247 | -0.01755 0.7837 247 | -0.02248 0.7252 247 | -0.01275 0.8419 247 |
| PCB 66 | 0.02672 0.6705 256 | 0.01791 0.7773 252 | -0.00352 0.9552 256 | 0.04711 0.4530 256 | -0.00198 0.9748 256 | -0.00581 0.9263 256 | -0.01168 0.8525 256 | 0.00109 0.9862 256 |
| PCB 74 | **0.12433 0.0469 256** | **0.13069 0.0382 252** | 0.11951 0.0562 256 | **0.17190 0.0058 256** | 0.12251 0.0502 256 | 0.10261 0.1014 256 | 0.11040 0.0779 256 | 0.11900 0.0572 256 |
| PCB 99 | **0.40506 <.0001 253** | **0.38234 <.0001 249** | **0.37445 <.0001 253** | **0.39275 <.0001 253** | **0.41353 <.0001 253** | **0.34853 <.0001 253** | **0.36137 <.0001 253** | **0.36251 <.0001 253** |
| PCB 101 | **0.55785 <.0001 256** | **0.51275 <.0001 252** | **0.23331 0.0002 256** | **0.36742 <.0001 256** | **0.36203 <.0001 256** | **0.29803 <.0001 256** | 0.09361 0.1353 256 | 0.01612 0.7975 256 |
| PCB 105 | **0.30247 <.0001 256** | **0.33106 <.0001 252** | **0.26742 <.0001 256** | **0.30079 <.0001 256** | **0.31065 <.0001 256** | **0.25276 <.0001 256** | **0.31300 <.0001 256** | **0.32349 <.0001 256** |
| PCB 110 | **0.29318 <.0001 253** | **0.30598 <.0001 249** | **0.12403 0.0488 253** | **0.23966 0.0001 253** | **0.20292 0.0012 253** | **0.17066 0.0065 253** | 0.08096 0.1993 253 | 0.00913 0.8851 253 |
| PCB 114 | **0.29380 <.0001 256** | **0.31415 <.0001 252** | **0.34810 <.0001 256** | **0.38529 <.0001 256** | **0.28534 <.0001 256** | **0.30954 <.0001 256** | **0.28519 <.0001 256** | **0.35314 <.0001 256** |
| PCB 118 | **0.39742 <.0001 256** | **0.42017 <.0001 252** | **0.35712 <.0001 256** | **0.39241 <.0001 256** | **0.39908 <.0001 256** | **0.33482 <.0001 256** | **0.36511 <.0001 256** | **0.39126 <.0001 256** |
| PCB 138 | **0.76690 <.0001 256** | **0.74994 <.0001 252** | **0.61951 <.0001 256** | **0.68719 <.0001 256** | **0.66179 <.0001 256** | **0.56698 <.0001 256** | **0.43271 <.0001 256** | **0.47475 <.0001 256** |
| PCB 146 | **0.76013 <.0001 248** | **0.79361 <.0001 244** | **0.64927 <.0001 248** | **0.67093 <.0001 248** | **0.66722 <.0001 248** | **0.61694 <.0001 248** | **0.41769 <.0001 248** | **0.47606 <.0001 248** |
| PCB 153 | **0.81400 <.0001 256** | **0.82614 <.0001 252** | **0.70233 <.0001 256** | **0.72964 <.0001 256** | **0.71700 <.0001 256** | **0.63567 <.0001 256** | **0.45919 <.0001 256** | **0.49010 <.0001 256** |
| PCB 156 | **0.45578 <.0001 256** | **0.52653 <.0001 252** | **0.62922 <.0001 256** | **0.55873 <.0001 256** | **0.51481 <.0001 256** | **0.49905 <.0001 256** | **0.39992 <.0001 256** | **0.44933 <.0001 256** |
| PCB 157 | **0.28879 <.0001 256** | **0.34550 <.0001 252** | **0.50634 <.0001 256** | **0.43339 <.0001 256** | **0.37424 <.0001 256** | **0.38480 <.0001 256** | **0.35283 <.0001 256** | **0.42005 <.0001 256** |
| PCB 167 | **0.58476 <.0001 256** | **0.57267 <.0001 252** | **0.50016 <.0001 256** | **0.57171 <.0001 256** | **0.50116 <.0001 256** | **0.44905 <.0001 256** | **0.34739 <.0001 256** | **0.46141 <.0001 256** |
| PCB 170 | **0.77873 <.0001 256** | **0.80718 <.0001 252** | **0.79875 <.0001 256** | **0.76890 <.0001 256** | **0.75578 <.0001 256** | **0.67254 <.0001 256** | **0.48761 <.0001 256** | **0.53190 <.0001 256** |
| PCB 172 | **0.76039 <.0001 256** | **0.78991 <.0001 252** | **0.69525 <.0001 256** | **0.68386 <.0001 256** | **0.71270 <.0001 256** | **0.65277 <.0001 256** | **0.43292 <.0001 256** | **0.48239 <.0001 256** |
| PCB 177 | **0.93449 <.0001 256** | **0.91163 <.0001 252** | **0.56865 <.0001 256** | **0.67168 <.0001 256** | **0.71211 <.0001 256** | **0.61866 <.0001 256** | **0.34311 <.0001 256** | **0.26077 <.0001 256** |
| PCB 178 | **0.82340 <.0001 256** | **0.86416 <.0001 252** | **0.66439 <.0001 256** | **0.71499 <.0001 256** | **0.72223 <.0001 256** | **0.66995 <.0001 256** | **0.41657 <.0001 256** | **0.41654 <.0001 256** |
| PCB 180 | **0.80948 <.0001 256** | **0.86912 <.0001 252** | **0.85437 <.0001 256** | **0.80513 <.0001 256** | **0.83310 <.0001 256** | **0.76831 <.0001 256** | **0.56730 <.0001 256** | **0.55623 <.0001 256** |
| PCB 183 |  | **0.90592 <.0001 252** | **0.60831 <.0001 256** | **0.70499 <.0001 256** | **0.74104 <.0001 256** | **0.63847 <.0001 256** | **0.38830 <.0001 256** | **0.33065 <.0001 256** |
| PCB 187 | **0.90592 <.0001 252** |  | **0.73254 <.0001 252** | **0.75550 <.0001 252** | **0.83729 <.0001 252** | **0.78178 <.0001 252** | **0.52385 <.0001 252** | **0.42382 <.0001 252** |
| PCB 194 | **0.60831 <.0001 256** | **0.73254 <.0001 252** |  | **0.76774 <.0001 256** | **0.89601 <.0001 256** | **0.89571 <.0001 256** | **0.79777 <.0001 256** | **0.65272 <.0001 256** |
| PCB 195 | **0.70499 <.0001 256** | **0.75550 <.0001 252** | **0.76774 <.0001 256** |  | **0.78273 <.0001 256** | **0.74182 <.0001 256** | **0.62600 <.0001 256** | **0.50463 <.0001 256** |
| PCB 196 | **0.74104 <.0001 256** | **0.83729 <.0001 252** | **0.89601 <.0001 256** | **0.78273 <.0001 256** |  | **0.94365 <.0001 256** | **0.82499 <.0001 256** | **0.56755 <.0001 256** |
| PCB 201 | **0.63847 <.0001 256** | **0.78178 <.0001 252** | **0.89571 <.0001 256** | **0.74182 <.0001 256** | **0.94365 <.0001 256** |  | **0.85344 <.0001 256** | **0.54157 <.0001 256** |
| PCB 206 | **0.38830 <.0001 256** | **0.52385 <.0001 252** | **0.79777 <.0001 256** | **0.62600 <.0001 256** | **0.82499 <.0001 256** | **0.85344 <.0001 256** |  | **0.65269 <.0001 256** |
| PCB 209 | **0.33065 <.0001 256** | **0.42382 <.0001 252** | **0.65272 <.0001 256** | **0.50463 <.0001 256** | **0.56755 <.0001 256** | **0.54157 <.0001 256** | **0.65269 <.0001 256** |  |
